# Imbalance of SMC1 and SMC3 Cohesins Causes Specific and Distinct Effects

**DOI:** 10.1371/journal.pone.0065149

**Published:** 2013-06-12

**Authors:** Magdalena Laugsch, Jochen Seebach, Hans Schnittler, Rolf Jessberger

**Affiliations:** 1 Institute of Physiological Chemistry, Medical Faculty Carl Gustav Carus, Dresden University of Technology, Dresden, Germany; 2 Dept. of Anatomy and Vascular Biology, Westfälische Wilhelms-Universität Münster, Münster, Germany; Duke University, United States of America

## Abstract

SMC1 and SMC3 form a high-affinity heterodimer, which provides an open backbone of the cohesin ring, to be closed by a kleisin protein. RNAi mediated knock-down of either one heterodimer partner, SMC1 or SMC3, is expected to cause very similar if not identical phenotypes. However, we observed highly distinct, protein-specific phenotypes. Upon knock-down of human SMC1, much of SMC3 remains stable, accumulates in the cytoplasm and does not associate with other cohesin proteins. Most of the excess nuclear SMC3 is highly mobile and not or only weakly chromosome-associated. In contrast, human SMC3 knock-down rendered SMC1 instable without cytoplasmic accumulation. As observed by differential protein extraction and in FRAP experiments the remaining SMC1 or SMC3 proteins in the respective SMC1 or SMC3 knock-down experiments constituted a cohesin pool, which is associated with chromatin with highest affinity, likely the least expendable. Expression of bovine EGFP-SMC1 or mouse EGFP-SMC3 in human cells under conditions of human SMC1 or SMC3 knock-down rescued the respective phenotypes, but in untreated cells over-expressed exogenous SMC proteins mis-localized. Paucity of either one of the SMC proteins causes RAD21 degradation. These results argue for great caution in interpreting SMC1 and SMC3 RNAi or over-expression experiments. Under challenged conditions these two proteins unexpectedly behave differently, which may have biological consequences for regulation of cohesin-associated functions and for human cohesin pathologies.

## Introduction

The highly conserved nuclear protein complex cohesin is required for sister chromatid cohesion, and is involved in DNA repair and regulation of gene expression (for reviews see [1], [2], [3], [4], [5], [6], [7], [8]). Cohesin is composed of two Structural Maintenance of Chromosomes proteins, SMC1 and SMC3, and a kleisin protein like RAD21. This tripartite complex associates with other proteins, including a HEAT repeat protein such as SA1 or SA2. SMC1 and SMC3 carry a globular ATPase head domain, formed by the C- and N-termini of one SMC protein, which folds back onto itself. The N- and C-termini are linked by an extended coiled-coil region, which is interrupted by a central hinge domain through which SMC1 and SMC3 heterodimerize. The cohesin ring is closed by the kleisin, which binds with its N- and C-terminus to the head domains of SMC3 and SMC1, respectively.

Upon dimerization the hinge domains form a characteristic doughnut-like structure with two interaction surfaces separated by a central hole [9], [10]. Hinge domains contribute to formation of a V-shaped SMC heterodimer [10], [11], [12], and may be a target of regulatory factors [13], [14]. Hinge-mediated heterodimerization of SMC1 and SMC3 appears to be very stable, for isolated hinge dimers resist high salt and considerable detergent such as 0.01% SDS [15]. Yet, hinge-hinge interactions are dynamic as the dimers may open and form an entry gate for DNA [4], [9], [16], [17]. SMC3 acetylation is thought to induce conformational changes of the dimeric hinge structure during S phase and to allow dimer opening and loading of DNA through a positively charged channel within the “doughnut” [18].

In cells, SMC1 and SMC3 are believed to always exist as heterodimers in a one-to-one stoichiometry. Similarly, most prokaryotes contain only a single smc gene and its product forms homodimers [10], [19], [20], [21], [22]. The head domain of eukaryotic SMC1 also homodimerizes, speculated to reflect an evolutionary relict [23]. This association is less stable than hinge-mediated heterodimerization and it remains unclear, whether this interaction would bring two SMC proteins of the same kind together *in vivo* [24]. SMC1, SMC3 and RAD21 also interact each with itself. Dimer formation in eukaryotes depends on the presence of two different hinge regions, i.e. one each from SMC1 and SMC3. One intact hinge interaction interface is sufficient for heterodimerization, since replacement of three conserved glycine residues by alanine in either the SMC1 or the SMC3 hinge does not abolish *in vitro* hinge dimer formation, but mutating both hinge domains does [15]. At least in yeast specific mutations of either one of the hinge dimer interaction surfaces are lethal [24]. Mutated hinge domains still dimerize but in an altered manner with reduced stability. The residence time of such mutant cohesin complexes, which still form *in vivo*, on chromosomes was reduced [24]. Isolated dimeric hinge domains bind DNA *in vitro* [15], [25]. In hinge domain mutants, which reflect mutations seen in Cornelia de Lange Syndrome (CdLS) patients, DNA binding is increased and the cells became hypersensitive to irradiation [26].

In vertebrates a fraction of cohesin is bound to chromosomes at all phases of the cell cycle except between anaphase onset and telophase. Loading of cohesin in telophase requires several proteins including SCC2- and SCC4-type proteins. Cohesion is regulated by cohesion-promoting factors sororin and ESCO1/ESCO2 and cohesion-weakening factors WAPL, PDS5 and a histone deacetylase (reviewed in: [4], [8], [27]. The histone deacetylase was recently identified as HDCA8 [28]. The dissociation of cohesin from chromosomes involves two pathways. A large fraction, estimated at least 90%, of cohesin is removed from the chromosomes arms in prophase and prometaphase, triggerd by PLK1 mediated phosphorylation of SA2. WAPL and sororin also regulate the prophase removal pathway. Some cohesin remains associated with centromeres and on chromosome arms and is removed by separase cleavage of RAD21 at the meta-anaphase transition. Reassociation of cohesin, probably cohesin released during prophase, to chromosomes occurs already during telophase. This pool of reversibly bound cohesin, which has a mean residence time of about 25 min on chromatin, has likely important functions regulating transcription and the chromatin structure throughout interphase [2], [3], [29]. During S/G2 phase a second population of cohesin binds much more stably to chromatin. Soluble cohesin proteins not associated with chromatin probably also exist in association with each other [30] and such soluble cohesin may constitute a cohesin reservoire.

Several human pathologies are associated with failures in the “cohesin system”. These pathologies include the above mentioned CdLS, which features mutations in cohesin proteins SMC1 or SMC3, or in cohesin-associated factors such as the loading factor SCC2 (NIPBL) (reviewed in [31], [32], [33]) or the histone deacetylase HDAC8 [28]. Other cohesin-related human diseases include human cancer [34].

It is generally assumed that the SMC1/SMC3 heterodimer acts as a functional unit, and that elimination or large disruption of either of the two SMC proteins would cause the same or very similar phenotypes. While this may be true for the essential function of cohesin in sister chromatid cohesion it is not clear, which fate the partner SMC protein experiences if one of the SMC proteins is reduced. To perform their known biological functions, SMC1 and SMC3 likely need to be balanced and need to be localized to the nucleus, need to heterodimerize, and need to generate the different cohesin pools. Aberrant cell behavior or pathologies may result if any of these requirements is not met, which could occur naturally like in cohesin-related pathologies or in experimental settings such as RNA interference approaches. Therefore, we set out to determine the consequences of manipulating the balance between SMC1 and SMC3 and observed contrasting, protein-specific effects.

## Materials and Methods

### Cell culture conditions and drug treatment

HeLa cells, kindly provided by Dr. Frank Buchholz (MPI, Dresden, Germany), were maintained in Dulbecco's modified Eagle's medium (D-MEM) supplemented with 10% fetal calf serum (FCS; InVitrogen/Gibco), penicillin and streptomycin in a humidified atmosphere containing 5% CO_2_ in air. The EGFP-msSMC1 or EGFP-msSMC3 stably expressing HeLa cell lines were kindly provided by Dr. Ina Poser (MPI, Dresden, Germany). Leptomycine B (LMB; Sigma) was used at 5 µg/mL and added for 2 h prior to cell harvesting.

### DNA transfection and RNA interference

SMC1 is synonymous for SMC1A (SMC1α) in this communcation. For transient knock-down of SMC1 or EGFP-tagged proteins HeLa cells were transfected with esiEGFP or esiSMC1 RNA (kindly provided by Dr. Frank Buchholz) with Oligofectamine (Invitrogen). One ml of a 750 ng/mL of an esiRNA solution per one million cells was used unless specified otherwise. Alternatively, one mL of 50 pmol/mL of siSMC1, siSMC3 [34] or non targeting siRNA solution [35] was used. The cells were harvested 72 h post transfection unless indicated otherwise. The bovine SMC1 (bSMC1) [36] was subcloned into the BglI/BamHI site of pEGFP-C1. For transient DNA transfection 400,000 cells/mL were transfected with 1 µg/mL EGFP-bSMC1, or untagged bSMC1 in pCAGGS [37], or empty vector pEGFP-C1 (EGFPempty) using Lipofectamine 2000.

### Antibodies

Rabbit polyclonal antibodies against SMC1, SMC3, RAD21, TopoII, were purchased from Bethyl Laboratories Inc. The monoclonal mouse IgG1 ß-tubulin was from Sigma Inc. Rabbit polyclonal anti-karypoherin-ß1 and anti-cPLA2 purchased from Cell Signaling Technology. Goat polyclonal anti-EGFP antibody was obtained from the MPI-CBG, Dresden, Germany. The HRP conjugated secondary antibodies anti-goat, anti-rabbit or anti-mouse used for immunoblotting detection were purchased from Jackson Laboratory. Anti-mouse IgG1Alexa Fluor 488, anti-goat Alexa 488 and anti-rabbit Alexa Fluor 555 used for immunofluorescence were from InVitrogen and anti-mouse IgG2a FITC was from Southern Biotech.

### Cell lysates and western blot analysis

Cells for total protein extracts were harvested, washed twice with PBS and lysed in RIPA buffer containing a protease inhibitor cocktail (Roche, Mannheim, Germany). After incubation on ice for 1 h the lysates were centrifuged at 14,000 g, 4°C, 15 min and supernatants were collected. Nuclear and cytoplasmic extracts were prepared from cells by detergent lysis. The cells were washed twice with PBS, and resuspended in hypotonic buffer A (10 mM TRIS pH 8.0, 1 mM EDTA, 100 mM NaCl) containing a protease inhibitor cocktail (Roche, Mannheim, Germany), 2 mM Na_3_VO_4_, 2 mM DTT, 1 mM NaF, 2 mM Na_2_S_2_O_5_ and 0.5 mM spermidine. The swollen cells were treated with 2 mM MgCl_2_ and 0.1% NP-40 for 5 min on ice, and twice vortexed for 10 and 15 s, with a 10 s interval. The cytoplasmic fraction was removed by centrifugation at 4,000 rpm, 4°C, 3 min and the nuclei were washed with buffer A and centrifuged again. After resuspension in buffer B (10 mM TRIS pH 8.0, 1 mM EDTA) containing all supplements as described for buffer A, 250 mM ammonium sulfate was added and the lysates were incubated for 30 min on ice. Following ultra-centrifugation at 40,000 rpm at 4°C for 30 min the nuclear supernatant was collected. For a most stringent extraction, the pelleted nuclei were resuspended in RIPA buffer for additional 30 min at 4°C and supernatants were collected following centrifugation at 14,000 rpm and 4°C for 15 min.

Extractions with consecutively increasing concentrations of ammonium sulfate (AS) were performed by resuspending the nuclear pellets in buffer B without any AS (0 mM). After 10 min incubation and centrifugation at 8,000 rpm at 4°C for 5 minutes the supernatant was collected. The pellets were washed with buffer B without AS and resuspended in buffer B containing 50 mM AS and incubated for 10 min on ice. The supernatants (50 mM nuclear extracts) were collected by centrifugation at 8,000 rpm, 4°C, 5 min. After washing the nuclei with buffer B without AS, the pellets were resuspended in buffer B with 250 mM AS and incubated on ice for 10 min. Supernatants were collected after ultra-centrifugation at 40,000 rpm at 4°C for 30 min. For the final extraction the remaining pellets were resuspended in RIPA buffer for additional 30 min on ice and supernatants were collected following ultra-centrifugation at 40,000 rpm and 4°C for 30 min. Optionally, supernatants were subjected to immunoprecipitation (0.1 to 1 mg lysate protein, 50 µL protein A or G agarose beads (Invitrogen) with the indicated antibodies. Lysates or precipitated proteins were analysed after gel electrophoresis by IB using Hybond membranes (Amersham). For detection chemiluminescence (Pierce, ECL Western Blotting Substrate) was used. In some instances, the protein gels were silver-stained.

### Immunofluorescence microscopy

Cells cultured on cover slips were fixed with 3.7% formaldehyde for 15 min, incubated with 0.5% Triton X-100 in PBS for 10 min and with 3% fetal calf serum (FCS) for 1 h at RT. Primary antibodies, diluted in 1% FCS/PBS were incubated for 1 h at RT. Secondary antibodies were added for 30 min at 37°C diluted 1% FCS/PBS. The cells were permanently mounted in FluormountG (SouthernBiotech) for fluorescence microscopy (Axiophot, Zeiss) and analysed by its relative software (Axiovision AxiVs40 V 4.6.3.0; Zeiss). To produce the final figures, the images were transferred to Photoshop CS2 (Adobe Systems, San Jose, CA). Identical illumination and camera settings were used within each data set.

### mRNA isolation and PCR

The mRNA was isolated using the RNeasy Mini Kit (Qiagen) including DNAse digestion using RNase-free DNAse Set (Qiagen). The cDNA was generated by Superscript II Reverse Transcriptase (InVitrogen). Primer sequences for Smc1 were: forward 5′-CAAGTTCGAGAGCAAAGCGG-3′ and reverse 5′-TTCCTCCCAGAAACACACCAAG-3′, for Smc3 forward 5′-GGAGGGCAGTCAGTCTCAAG-3′, reverse 5′-AGCAAGGGCTACCAAGGATT-3′ and for Rad21 forward 5′-CAAATTGACCCAGAGCCTGT-3′, reverse 5′-CCCTGATGCATCTTCATCCT-3′. The cDNA was quantified by real-time qRT-PCR using QuantiTect SYBR Green PCR Kit (QIAGEN). For normalization of cDNA data, the ß-actin gene product was used with the primer sequences: forward 5′- CTCTTCCAGCCTTCCTTCCTG -3′ and reverse 5′- AGCACTGTGTTGGCGTACAG -3′. All oligonucleotides were from Eurofins MWG Operon (Ebersberg, Germany). Expression levels were calculated using Rotor Gene software 3000 (Corbet Research Inc.) and related to the mock or control-treated cultures. Melting point curve analysis resulting in homogenous signals verified the specificity of the primer. In addition, cDNA was analysed by PCR using the same primer pairs and conventional PCR and Dream Taq Polymerase (Fermentas) following agarose gel electrophoresis.

### Cell cycle analysis

The cells were harvested, washed twice with PBS and fixed in 70% Ethanol at −20°C. After rehydration and blocking in PBS containing 2% FCS and 2 mM EDTA the cells were incubated with 100 ng/mL RNAse at RT. The cells were then stained with propidium iodine (Sigma-Aldrich) at 50 ng/mL and the cell cycle status was analyzed by flow cytometry on an LSRII analyzer (BD Bioscience).

### FRAP

FRAP Measurements and analyses were performed as described previously [38] with few modifications. Cell cultures and siRNA/esiRNA tretament was done as described above. FRAP experiments were performed with a Leica TCS SP5 using the 488 nm laser line of an Argon laser (3.01 mW), a HeNe-Laser at 594 nm (0.59 mW) and a 405 nm Laser Diode (4.63 mW) at 37°. For acquiring the pre- and post-bleaching images the 488 nm Line was used with 10% power. A circular ROI with a diameter of 6 µm in the nucleus was selected and bleached for 1.25 s with maximal power from all lasers. 10 images were acquired before bleaching and 250 images were continuously acquired afterwards for further 80 s. To quantitatively analyze the fluorescence recovery process, background fluorescence was subtracted from all images, and the fluorescence of the bleached ROI was normalized to the total fluorescence intensity of the whole nucleus. The resulting curve for the normalized intensity I(t) was fitted with a Levenberg-Marquardt-algorithm to the following exponential function (1) (using a self-written LabView-Program) with the fitting parameters M_f_, τ and I_0_: (1) I(t) = M_f_−(M_f_ −I_0_) (exp(−t/τ)), where M_f_ is the mobile fraction, τ the time constant for the recovery process and I_0_ is the normalized fluorescence intensity of the ROI immediately after bleaching.

### Statistical analysis

IB images, analyzed by ImageJ (Version 1.42a, standard plug-ins) and the resulting values or values obtained by real-time PCR and eppendorf vapo protect (Eppendorf, Hamburg, Germany) were transferred to Microsoft Excel. The statistical analysis was carried out using the two-tailed paired t test and p values <0.05 were considered to be statistically significant (*).

### Proliferation assay

Living cells used for proliferation assays were stained with 10 µM CFSE (Cell Trace CFSE cell proliferation kit, Molecular Probes) for 15 minutes at 37°C in culture medium and further processed according to the manufacturers protocol. The proliferation status was analyzed at indicated time points by flow cytometry (BD Bioscience).

## Results

### SMC1 knock-down causes cytoplasmic mis-localization of SMC3

**Figure 1:**
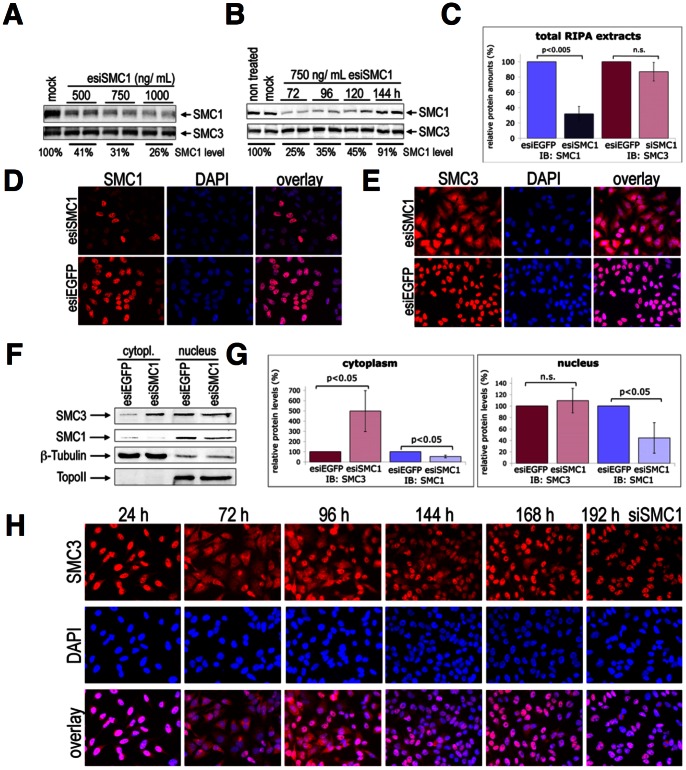
Transient down-regulation of endogenous human SMC1 in HeLa cells using specific esiRNA or siRNA impairs the nuclear localization of SMC3. (**A**) RIPA total cell extracts were prepared 72 h after treatment with three different concentrations of esiSMC1 and examined by IB using anti-SMC1 antibody. Mock transfected cells were used as negative control. The membrane was reprobed with anti-SMC3 antibody to confirm the specificity of esiSMC1 and equal loading. The percentages of SMC1 protein levels, normalized to SMC3 protein levels with respect to mock control set at 100%, are indicated. (**B**) Kinetics of recovery of SMC1 expression after treatment of cells with 750 ng/mL of esiSMC1 was analyzed by IB as described in A. (**C**) Quantification of SMC1 and SMC3 in RIPA total extracts of cells treated with 750 ng/mL of esiRNA and collected 72 h post transfection. Average of six independent experiments is shown. (**D**) IF microscopic analysis of SMC1 knockdown 72 h post esiRNA transfection by anti-SMC1 staining in red and DAPI in blue. Specific esiRNA against EGFP (esiEGFP) was used as a control. (**E**) IF microscopic analyses of esiSMC1- or esiEGFP-treated cells (72 h) using anti-SMC3 (red) and DAPI (blue). (**F**) Cytoplasmic and nuclear extracts from esiSMC1 or control treated cells were analyzed by IB using anti-SMC3. The membrane was reprobed with anti-SMC1. Anti-ß tubulin and Topo II antibodies were used to determine the purity of nuclear and cytoplasm extracts. (**G**) Quantification of results from four independent experiments that were performed as described in F. (**H**) Time course of SMC3 localization upon treatment of cells with siSMC1 (#1) as visualized by IF microscopy using anti-SMC3 (in red) and DAPI (in blue).

To examine the fate of SMC3 that largely lacks its SMC1 heterodimerization partner, esiRNA [39] was used to transiently knock-down human SMC1 (“esiSMC1”). To establish an effective esiRNA concentration and the kinetics of its effect, total cell extracts from HeLa cells treated once for 72 h with different concentrations of esiSMC1 (500, 750 and 1000 ng/mL per 1×10^6^ cells) were analyzed by immuno blotting (IB) using anti-SMC1 antibody (Fig. 1A). SMC1 protein level was not affected in mock treated cells. At 750 ng/mL transfected esiSMC1 an SMC1 knockdown to 31% was observed and was used in subsequent experiments. A decrease in SMC1 protein levels was observed starting 24 h after a single transfection (Fig. S1A) and maximal SMC1 protein reduction to 16% was observed at 72 h (Fig. 1B and S1A). At 144 h after transfection, SMC1 has recovered to levels close to those seen before treatment (Fig. 1B). At 750 ng/mL esiSMC1 and the 72 h time point, SMC1 protein was in average reduced to 30% (±10%) compared to cells treated with esiEGFP esiRNA (Fig. 1C; n = 7). At a transfection efficiency of about 80%, SMC1 levels in the transfected cells are certainly below the 30% mark, which included the 20% non-transfected cells. SMC3 protein levels remained largely unchanged upon SMC1 knock-down (Fig. 1A–C) – a small decrease occasionally observed was not statistically significant – demonstrating the specificity of esiSMC1 treatment and suggesting stability of SMC3 even in absence of SMC1. Immunofluorescence (IF) analyses of SMC1 after esiSMC1 treatment showed very weak nuclear signals in most of the cells (Fig. 1D). Some non-transfected cells are present and show strong nuclear SMC1 staining, which serves as positive staining control. No cytoplasmic staining was observed. Treatment with control esiEGFP showed no effect on SMC1 staining (Fig. 1D).

**Figure 2:**
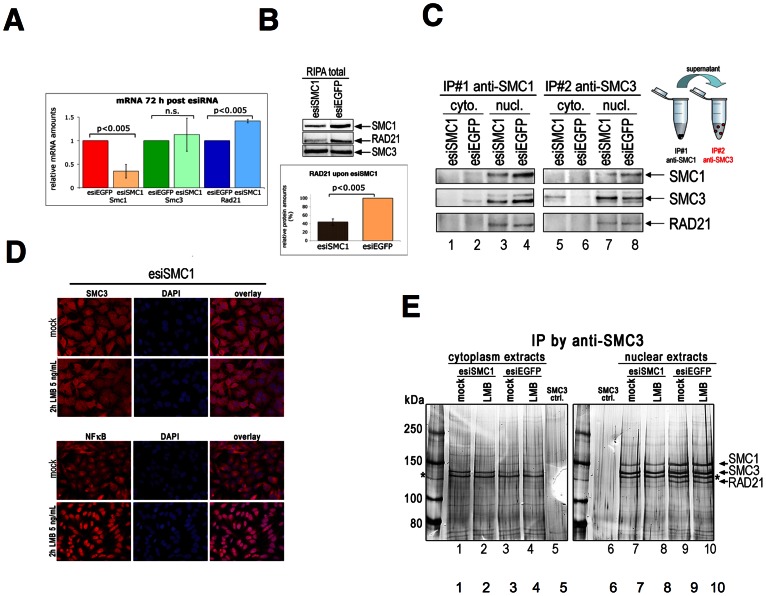
Characterization of cytoplasmic SMC3 protein. (**A**) Expression of mRNA of Smc1, Smc3 and Rad21 72 h after esiSMC1 treatment, compared to control (esiEGFP), was examined by real-time RT-PCR. Relative quantification of gene expression was achieved by normalization to ß-actin, (n = 3). (**B**) Effect of SMC1 knockdown on RAD21 protein in total RIPA extracts as monitored by IB with anti-Rad21 antibody 72 h post esiSMC1 or esiEGFP transfection. Bottom: quantification of IB from three independent experiments. (**C**) Sequential IP. IP #1 from nuclear and cytoplasmic extracts from esiSMC1-treated and control cells. The supernatant was used for IP#2 with anti-SMC3 antibody. Eluates were analyzed by IB using anti-RAD21, -SMC3, and -SMC1 antibodies. (**D**) Nuclear export was inhibited 70 h after esiSMC1 or control treatment by addition of LMB to a final concentration of 5 ng/mL for another 2 h. The localization of SMC3 (in red, top half) was examined by immunofluorescence. NFκB (in red, bottom half) was used to confirm the LMB effect. DNA was visualized by DAPI (in blue). (**E**) Immunoprecipitation of SMC3 from cytoplasm and nuclear extracts after LMB inhibition was performed and eluates examined by silver staining. Arrows indicate the positions of SMC1, SMC3 and RAD21. The asterisk indicates an unspecific band as specified by mass spectrometric analysis.

Neither measurable changes in proliferation and cell cycle progression (Fig. S1B, S1C) nor increased sensitivity to DNA damaging drugs such as mitomycine C (MMC) (Fig. S1D) were observed in cells treated with esiSMC1 as described above. The kinetics of ATM catalyzed phosphorylation of H2AX to γH2AX, or of SMC1 at S-957 [40], [41], the formation of SMC6 foci, and levels of apoptosis remained normal (Fig. 1D, 1E, and data not shown), as do long-term cell cycle profiles (Fig. 2A). This indicates that for these core functions of cohesin, less than 30% of cohesin is required.

Since total protein levels of SMC3 remained largely unchanged upon SMC1 knockdown, we investigated the intracellular localization of SMC3 after esiSMC1 or esiEGFP transfection. Surprisingly, upon SMC1 knockdown a large portion of SMC3 appeared in the cytoplasm, while its nuclear staining remained unchanged (Fig. 1E–H). Control treatments with esiEGFP did not alter the distribution of SMC3 (Fig. 1E–G). IB of cytoplasmic and nuclear extracts confirmed the IF data and showed a strong increase in cytoplasmic SMC3 specifically upon SMC1 knockdown. A very weak signal for SMC1 and SMC3 in the cytoplasmic extracts in control cells very likely results from unavoidable nuclear protein contamination (Fig. 1F). In average the cytoplasmic SMC3 protein level was increased to 500% (±200%) over the basal (background) level (100%) of control treated cells (n = 4). Only a slight, if any, decrease of nuclear SMC3 was observed in esiSMC1 treated cells (Fig. 1F). The nuclear SMC1 protein level in esiSMC1 treated cells was significantly reduced to 40% (±20%) of mock treated or untreated cells (100%) (Fig. 1G). Two different, independent siRNA sequences specific for human SMC1 were used and yielded very similar results (Fig. 1H and Fig. S2B). The kinetics of cytoplasmic SMC3 localization following siSMC1 treatment follows the pattern seen in esiSMC1 treatment: strong cytoplasmic signals for SMC3 at 72 h post treatment, and decrease of cytoplasmic staining starting around 96 h and eventually full recovery of nuclear staining at 192 h (Fig. 1H).

These data indicate the continued presence of a small fraction of nuclear cohesin, probably required to maintain essential functions, and a large cytoplasmic accumulation of SMC3 when its partner SMC1 is mostly absent.

### Does cytoplasmic SMC3 form cohesin complexes?

One explanation for the appearance of cytoplasmic SMC3 could be aberrant up-regulation of *Smc3* gene expression in response to SMC1 knock-down. Hence, the mRNA expression of *Smc1*, *Smc3* and *Rad21* was examined by real-time RT-PCR (Fig. 2A). Relative quantification was achieved by normalization to ß-actin and comparison to esiEGFP treated cells. The data confirm reduction of *Smc1* mRNA after esiSMC1 treatment and show unaltered *Smc3* gene expression. A significant increase of *Rad21* gene expression was observed, possibly in an attempt by the cell to complement loss of RAD21, since levels of RAD21 protein were reduced to 40% (±10%) in esiSMC1 treated cells (Fig. 2B). Thus, the reduction of SMC1 affects not only SMC3 localization, but also the presence of RAD21 protein, which in absence of sufficient SMC1/SMC3 partners is probably degraded.

Cytoplasmic SMC3 that appears upon SMC1 knock-down may exist without associated cohesin subunits – there is certainly not sufficient SMC1 available for dimerization. Two consecutive rounds of immuno precipitation (IP) were used to determine the molecular status of the excess cytoplasmic SMC3. Residual cytoplasmic and nuclear extracts used once for immuno precipitation (IP) with anti-SMC1 (IP #1) were used for a second IP (IP #2) with anti-SMC3 antibody. Precipitates were analyzed by IB (Fig. 2C). As expected no SMC3, SMC1 or RAD21 was detected in anti-SMC1 IP from cytoplasmic extracts, either in esiSMC1 (lane 1) and only very little, if any, in esiEGFP (lane 2) treated cells. The decreased SMC3 signal in anti-SMC1 IP from nuclear extracts from esiSMC1 treated cells (lane 3) confirms the strongly reduced presence of SMC1/SMC3 complexes in nuclei. Similarly, the SMC1 signal is strongly decreased in IPs from nuclear extract of esiSMC1 treated cells (lane 3 to compare with 4), as are levels of SMC3 and RAD21. Re-precipitation of the supernatants with anti-SMC3 showed that SMC3 is present without associated SMC1 or RAD21 in the cytoplasm of esiSMC1 treated cells (lane 5). No SMC3 is seen in the corresponding fraction from esiEGFP treated cells (lane 6). A large portion of SMC3 is still visible in anti-SMC3 re-precipitates from the nuclear extract supernatants of esiSMC1 treated, but less is precipitated from supernatants of esiEGFP treated cells (lane 7 and 8). This indicates that there is also some excess of SMC3 in the nuclei of esiSMC1-treated cells. This is consistent with the reduction of SMC1 and RAD21 in anti SMC3 re-precipitates from esiSMC1 treated cells.

The cytoplasmic mis-localization of SMC3 in absence of most SMC1 raises the question, whether SMC3 without its SMC1 partner was initially imported into the nucleus and then released in the cytoplasm or stayed in the cytoplasm without ever translocating into the nucleus. In the latter case, blocking of export of SMC3 from the nucleus would not reduce the cytoplasmic accumulation of SMC3 in esiSMC1 treated cells. Human, mouse and bovine SMC3 harbor a conserved leucine-rich nuclear exit signal (NES) at position 1023–1028. Nuclear export was inhibited by Leptomycine B (LMB), which was added for two hours at 70 h after esiSMC1 or esiEGFP treatment. The distribution of SMC3 examined by IF and was not visibly altered (Fig. 2D). For a positive control NFκB distribution after LMB treatment was assessed. NFκB strongly accumulated in the nucleus upon LMB treatment (Fig. 2D, bottom). Additionally, anti-SMC3 IP from cytoplasmic and nuclear extracts after esiSMC1 or esiEGFP treatment and LMB inhibition were examined by silver staining. No differences in signals were observed upon LMB treatment in SMC1, SMC3 or RAD21, neither in esiEGFP or esiSMC1 treated cells (Fig. 2E). Besides, no obvious differences were seen in SMC3 mobility in SDS-PAGE between cytoplasmic (lanes 1–4) and nuclear SMC3 (lanes 7–10), and thus there is no obvious indication for extensive posttranslational modifications of cytoplasmic SMC3 in SMC1 knock-down cells.

### Excess nuclear SMC3 barely associates with chromatin

**Figure 3:**
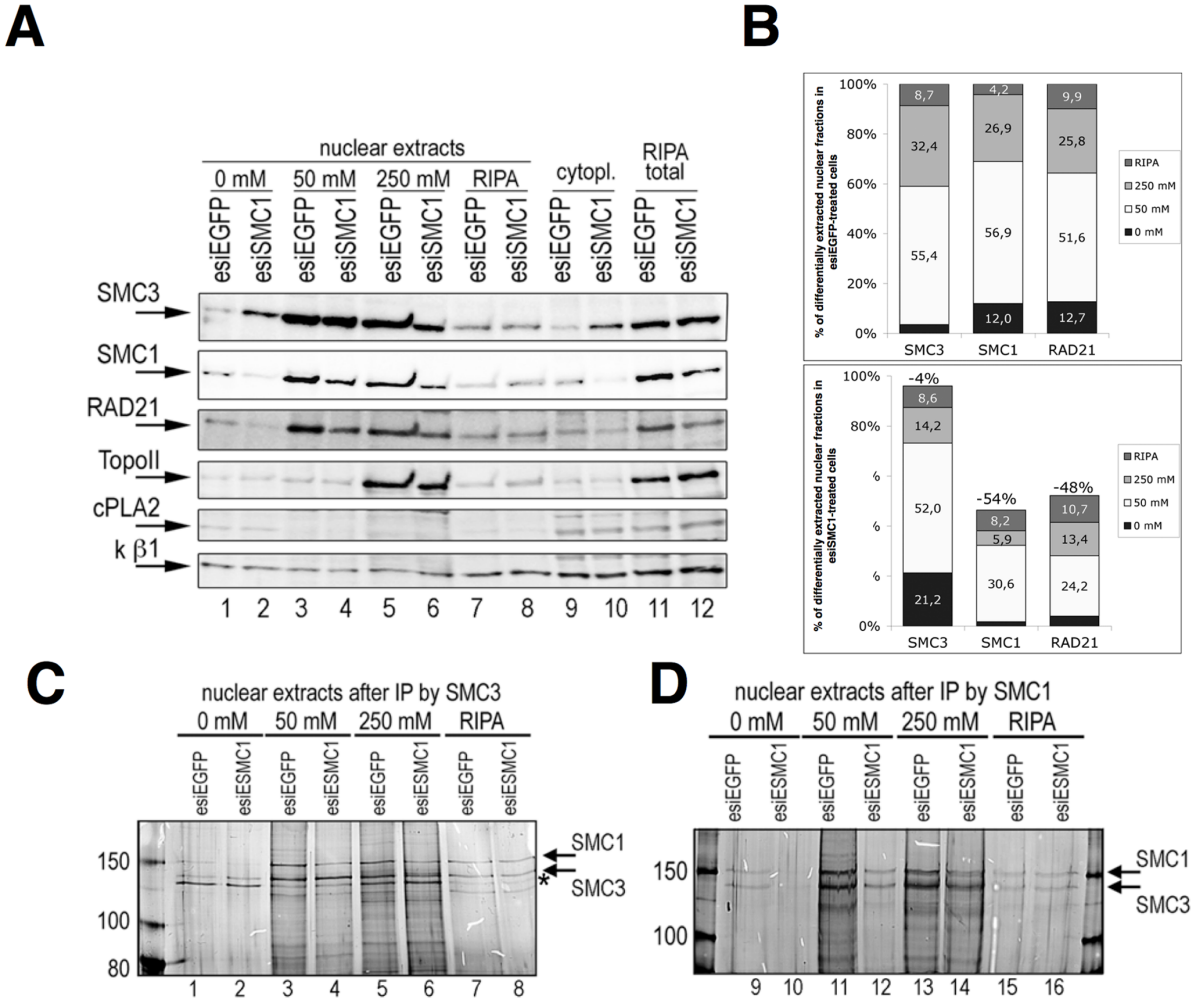
Differential salt extraction from control or esiSMC1-treated cells. Representative results from three independent experiments are shown. (**A**) Nuclear extracts from esiSMC1 and control treated cells were prepared by successive treatment with increasing salt (0 mM, 50 mM, 250 mM ammonium sulfate) concentrations. After the last salt extraction RIPA buffer was used for the final extraction step. IB analysis for SMC3, SMC1 and RAD21 followed. Topo II was used as nuclear loading control, cPLA2 as a cytoplasmic control, and for a loading control of all fractions karyopherin (k) ß1 was used. (**B**) Quantification of the relative distribution of SMC1, SMC1 and RAD21 in different nuclear fractions after esiEGFP (top) or esiSMC1 (bottom) treatment as presented in A. (**C**) IP with anti-SMC3 or (**D**) anti-SMC1 antibodies was done with all fractions and eluates were examined by silver staining. The asterisk indicates the unspecific band described in 2D.

The above data suggest the cytoplasmic SMC3 protein in esiSMC1 treated cells does not translocate to and from the nucleus. However, there is still nuclear SMC3 at levels that are unchanged upon SMC1 knock-down, although SMC1 and RAD21 are strongly reduced. For the lack of partners, this excess nuclear SMC3 is most likely not incorporated into cohesin complexes (Fig. 2C, lane 7), but does such excess nuclear SMC3 stably associate with chromatin? Nuclei from esiSMC1 and esiEGFP treated cells were extracted by three consecutive salt concentrations of 0 mM, 50 mM, and 250 mM ammonium sulfate (AS) to elute protein fractions with increasing affinity to chromatin (Fig. 3A, B). The remainder of the thrice-extracted nuclei was finally extracted with RIPA buffer. In controls, cytoplasmic and total extracts showed the cytoplasmic appearance of SMC3 as well as the reduction of SMC1 and RAD21 specifically in SMC1 knock-down cells (lanes 9–12). TopoII was used as a nuclear and total extract loading control (lanes 5,6 and 11,12) and cPLA2 as a cytoplasmic control (lanes 9,10) to assess the purity of nuclear extracts. As a loading control for all fractions karyopherin ß1 was used.

Only a small decrease of 4% of total SMC3 was seen upon SMC1 knockdown, but total amounts of SMC1 and RAD21 were decreased by 54% and 48% resp. as indicated in Fig. 3B, bottom graph, when compared to their levels in esiEGFP treated cells (100%). Only very little nuclear SMC3, SMC1 and RAD21 eluted at 0 mM AS from esiEGFP cells (lane 1). However, a much larger portion (21%) of nuclear SMC3, but not of SMC1 and RAD21, elutes at 0 mM after esiSMC1 treatment of the cells (lane 2). Correspondingly, the high salt-eluted fraction of SMC3 from esiSMC1 treated cells is reduced from 32% to 14%. Thus, a considerable pool of excess SMC3 does not or only very weakly bind chromatin if SMC1 is scarce. The disproportionally larger reduction of SMC1 and RAD21 (to 1.7% and 4% respectively) that elutes at 0 mM may indicate that the non chromatin-associated pool is the one that can be most easily reduced in esiSMC1-treated cells. Most SMC3, SMC1and RAD21 (>50%) elutes from the nuclei at 50 mM AS (lane 3) after esiEGFP (lane 3). In esiSMC1 treated cells this fraction of SMC3 is unchanged (52%). Of the remaining SMC1 and RAD21, 66% and 46%, respectively, elute at 50 mM (31% and 24% of the pre-treatment amounts), and thus also an unchanged proportion of these two cohesins elutes at 50 mM salt. Between 26% and 32% of SMC3, SMC1 or RAD21 eluted at 250 mM (lanes 5,6), and this fraction is generally decreased about 2-fold in esiSMC1-treated cells. However, the fraction that requires the most stringent extraction conditions, the RIPA fraction, is increased for SMC1 (4-fold from 4.2% to 18% of the remaining SMC1) and RAD21 (2-fold from 9.9% to 21% of the remaining RAD21), but not for SMC3.

These data demonstrate that much of the excess nuclear SMC3 does not or only very weakly associate with chromatin and supports the above conclusion of the non-cohesion role of excess SMC3 in both, the nucleus and the cytoplasm. The data also suggest that in esiSMC1-treated cells the small fraction of remaining cohesin including SMC1 and RAD21 associates with particular high affinity with chromatin.

Furthermore, anti-SMC1 and anti-SMC3 IPs were performed from all differentially extracted nuclear fractions and the eluates were examined by silver staining (Fig. 3C, D). Anti-SMC3 IP (Fig. 3C) from 0 mM AS extracts yielded weak SMC1 and SMC3 bands, but in very similar intensities from esiSMC1 as from esiEGFP treated cells (lane 1). The corresponding IP from esiSMC1 cell extracts (lane 2) shows almost only the SMC3 band, which is slightly stronger. A similar pattern from esiSMC1 cells is seen at 50 AS (lanes 3 and 4) as well as at 250 AS: weaker SMC1 and disproportionally stronger SMC3, which in the 250 mM AS extract shows two bands close to each other. The nature of these bands, possibly posttranslationally modified forms, may be determined in future experiments. No differences between esiEGFP and esiSMC1 were observed in RIPA fractions (lanes 7 and 8), where a 1∶1 SMC1/SMC3 pattern is seen, suggesting that this constitutes the remainder of the cohesin, which binds with highest affinity. As above, the prominent band appearing below SMC3 is unspecific. The results are in agreement with data above (Fig. 1E, F and 2F), which show continued presence of some excess SMC3 in the nucleus of SMC1 knock-down cells.

In accordance with Figure 3A, the anti-SMC1 IP (Fig. 3D) generated essentially no signal from the 0 mM AS esiSMC1 cell nuclear extract (lane 10), again indicating that the most loosely chromatin-associated fraction of SMC1 is absent under SMC1 knock-down conditions. In all subsequent extracts, an approximately 1∶1 ratio of SMC1 and SMC3 was observed (lanes 11–16) with the expected reduced SMC1/SMC3 signal upon SMC1 knock-down (lanes 12 and 14). No difference between control and esiSMC1 samples is seen in the RIPA sample (lanes 15 and 16), confirming the presence of a relatively small amount of the heterodimer in this most tightly chromatin-associated fraction. This suggests, the soluble, less tightly or not chromatin-associated pool of cohesin is the most expendable.

### Transiently over-expressed SMC1 remains cytoplasmic and is only marginally incorporated into cohesin

**Figure 4:**
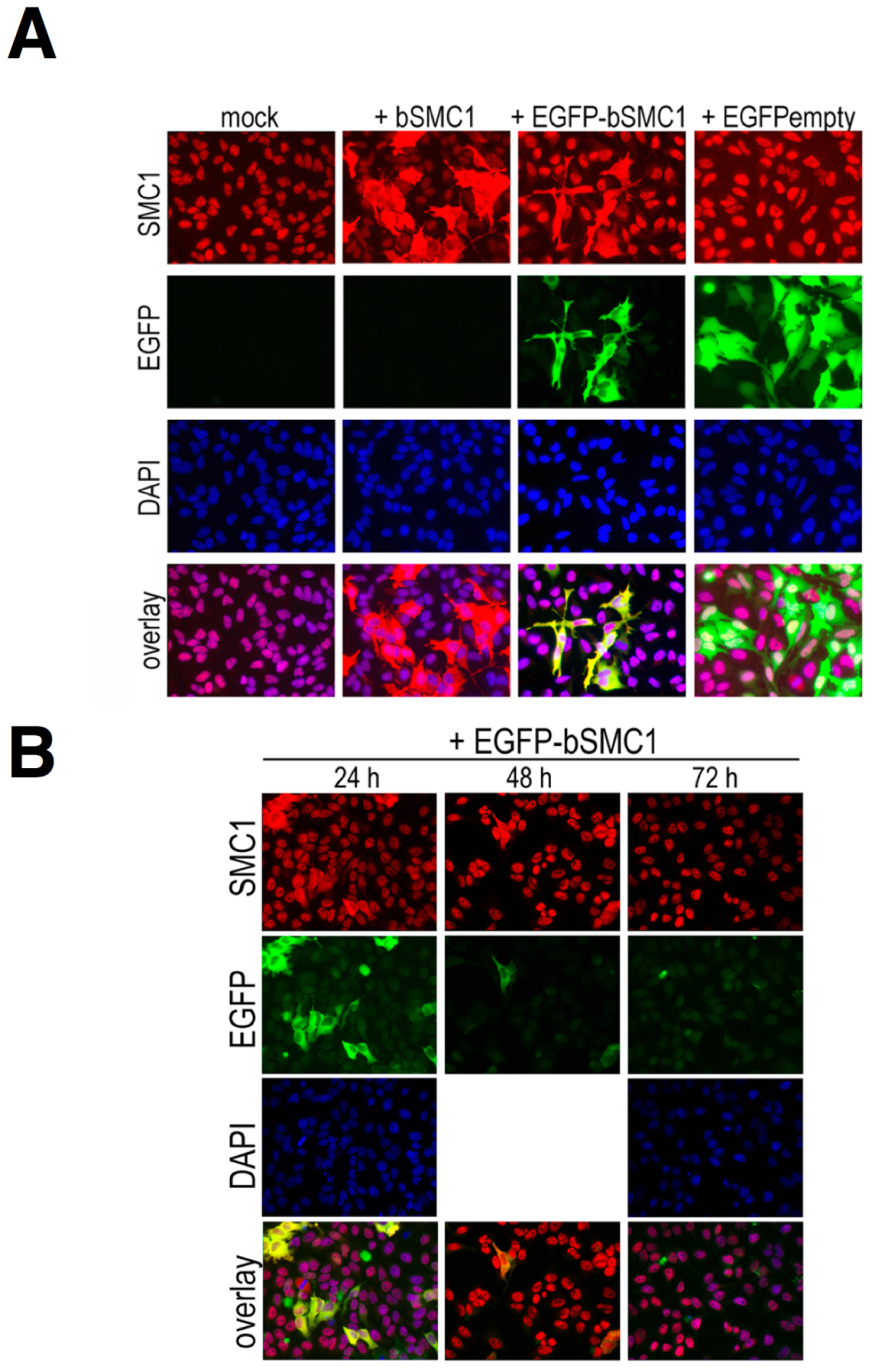
IF analysis of transiently over-expressed bovine SMC1 (EGFP-bSMC1) in HeLa cells. Anti-SMC1 antibody (red) and EGFP (green) were used. Nuclear staining was obtained using DAPI (blue) (**A**) 24 h after transfection of bovine SMC1 without any tag or N-terminally tagged EGFP-bSMC1. As a control an EGFP-empty vector was used. Anti-SMC1 antibody (red) stains both, tagged and non-tagged SMC1 proteins. (**B**) Stability of transiently overexpressed EGFP-bSMC1 (green) 24, 48 and 72 h after transfection.

The above experiments indicate the 1∶1 stoichiometry between SMC1 and SMC3 proteins is critical. Thus, over-expression of SMC1 or SMC3 may also elicit distinct phenotypes. SMC proteins are ubiquitous and evolutionary highly conserved and thus we used EGFP-bSMC1 (bovine), which is nearly identical to human SMC1 (99.8% amino acid identity) in a two-species-system, which allows further manipulations. The functionality of EGFP-bSMC1 in human cells is demonstrated in the following section. Transiently over-expressed EGFP-tagged like untagged bSMC1 largely failed to localize to HeLa cell nuclei, but rather strongly accumulated in the cytoplasm (Fig. 4A). Transfection of EGFP control vector (EGFPempty) did not alter the pattern of endogenous SMC1 staining. The stability of over-expressed EGFP-bSMC1 declines beyond 24 h after transfection and the protein is absent 72 h after transfection (Fig. 4B).

### Functional complementation of down-regulated hSMC1 by exogenous bSMC1 in a two-species system

**Figure 5:**
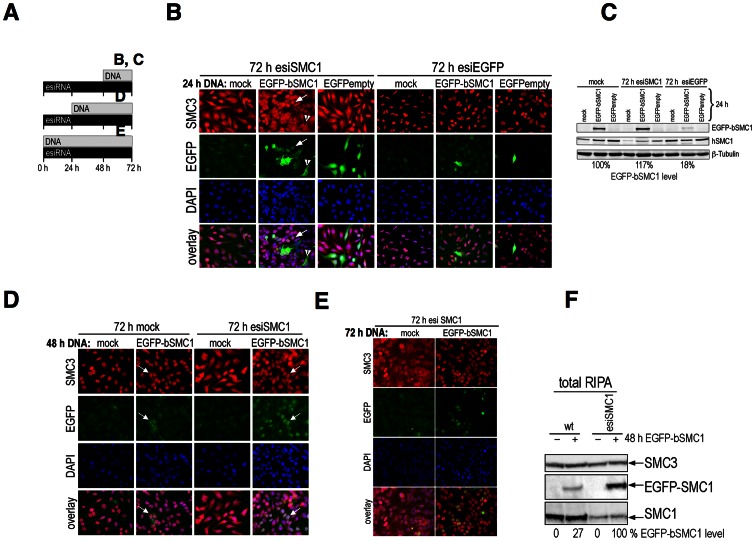
Two-species system: knockdown of human SMC1 by esiRNA and rescue by over-expression of bovine SMC1 (EGFP-bSMC1). (**A**) Scheme of esiSMC1 and plasmid DNA transfection. (**B**) Nuclear localization of EGFP-bSMC1 (green) and rescue of SMC3 mis-localization by anti-SMC3 staining (red) 72 h after esiSMC1 and 24 h EGFP-bSMC1. The arrows indicate nuclear localization (large arrow) of SMC3 and EGFP-bSMC1 (rescue) or cytoplasmic localization (arrowhead) for both (no rescue). The EGFP-empty vector (right) was used as control. (**C**) Using anti-EGFP antibody for IB analysis of total cell extracts (RIPA), the efficiency of EGFP-bSMC1 expression upon esiSMC1 and esiEGFP was monitored. The percentage of EGFP-bSMC1 levels was normalized to ß-tubulin as a loading control. The SMC1 knock-down was confirmed using anti-SMC1 antibody. (**D**) Nuclear localization of EGFP-bSMC1 (green) 48 h after transfection with EGFP-bSMC1 of esiSMC1-treated cells. Nuclear SMC3 is shown using anti-SMC3 antibody (red). The arrows indicate rare nuclear localization of EGFP-bSMC1 in cells not treated with esiSMC1 and much more nuclear EGFP-bSMC1 localization in cells treated with esiSMC1. (**E**) Rescue of SMC3 distribution seen with anti-SMC3 antibody (red) 72 h after co-transfection of esiSMC1 and EGFP-bSMC1. The DNA was visualized in each case using DAPI (blue). (**F**) Total cell extracts (RIPA) prepared from cells transfected for 48 h with EGFP-bSMC1 and 72 h esiSMC1 treatment. IB analyses used anti-SMC3, -EGFP or -SMC1 antibodies. Percentages of EGFP-bSMC1 expression are shown at the bottom.

Next we asked, whether EGFP-bSMC1 can functionally complement depletion of endogenous SMC1 after esiSMC1 treatment of HeLa cells. We exploited the divergence of the DNA and RNA sequences in this two-species system. The experimental scheme is shown in Figure 5A and indicates the different time points of DNA transfection after esiRNA treatment. The esiSMC1 reagent for human SMC1 does not affect EGFP-bSMC1 at any time point (Fig. 5B–E). The EGFP-bSMC1 or EGFP vectors were transfected into the cells for expression for 24, 48, or 72 h. EsiSMC1 was present throughout the 72 h period. At 24 h after EGFP-bSMC1 transfection and 72 h after SMC1 reduction, IF (Fig. 5B) and IB analyses (Fig. 5C) were performed. Functional complementation was achieved for both phenotypes in that increased nuclear localization of EGFP-bSMC1 and partial rescue of the cytoplasmic mis-localization of SMC3 were observed (Fig. 5B, left). The EGFP control vector did not produce any of these effects. The effectivity of esiRNA was confirmed using esiEGFP, which targets EGFP-bSMC1 or EGFP mRNA for degradation (Fig. 5B, right). The EGFP signal was in each case heavily reduced upon esiEGFP treatment (to 18% for EGFP-bSMC1), and endogenous SMC1 and SMC3 protein levels and distribution remained unchanged (Fig. 5B, C).

The EGFP-bSMC1 protein level was moderately increased to 117% if esiSMC1 was used (Fig. 5C), perhaps because more SMC3 was available to dimerize with EGFP-bSMC1 to stabilize it. Intense nuclear localization of EGFP-bSMC1 was seen 48 h after EGFP-bSMC1 transfection into esiSMC1 treated cells (Fig. 5D, fourth row). The relatively low percentage of green cells results from the limited efficiency of the consecutive double transfection of esiRNA and plasmid, but even cells less bright for the GFP signal may express low levels of the exogenous EGFP-bSMC1. The rescue of SMC3 nuclear localization was more pronounced at 48 h and 72 h than at 24 h after EGFP-bSMC1 transfection (compare Fig. 5B with 5D, 5E). However, in cells transfected with EGFP-bSMC1 but not with esiSMC1, most of the cytoplasmic EGFP-bSMC1 was degraded and barely visible in the cytoplasm or nucleus 48 h after transfection (Fig. 5D, second row). This is consistent with the data showing strong reduction of cytoplasmic EGFP-bSMC1 48 h and 72 h after plasmid transfection (Fig. 4B) and indicates that – different from excess SMC3 – excess SMC1 is not stable. Co-transfection of esiSMC1 and EGFP-bSMC1 almost completely rescued the nuclear localization of SMC3 (Fig. 5E; 72 h). The EGFP-bSMC1 signal was decreased when compared to the 48 h image and only detectable in the nucleus (Figure 5E).

Together this suggests that excess SMC3 supports nuclear localization of excess SMC1, that nuclear localization of SMC1 (in association with SMC3) prevents its degradation, and that excess SMC1 is instable.

Since the most intense nuclear EGFP-bSMC1 staining was seen 48 h after EGFP-bSMC1 transfection into cells that were treated for a total of 72 h with esiSMC1, we used these conditions for further analysis. Total cell extracts were obtained 72 h after esiSMC1 or mock treatment with or without expression of EGFP-bSMC1 for the final 48 h (“72/48 h cells” as in D). These total cell lysates (RIPA) were initially analysed by IB using anti-SMC3 and showed largely unchanged levels of SMC3 in protein in all samples (Fig. 5F). Reprobing the same membrane with anti-EGFP antibody revealed only a weak signal (27%) for EGFP-bSMC1 in cells not treated with esiSMC1 and a much stronger signal in esiSMC1 treated cells (100%). The data are consistent with the above IF results showing stabilization of EGFP-bSMC1 upon knock-down of endogenous SMC1. The knock-down of endogenous human SMC1 is not affected by expression of EGFP-bSMC1 (Fig. 5F; two right lanes, bottom blot). IP experiments showed complex formation of EGFP-bSMC1 with SMC3 in untreated and esiSMC1-treated cells (data not shown).

### SMC3 knock-down triggers degradation of SMC1 and RAD21

**Figure 6:**
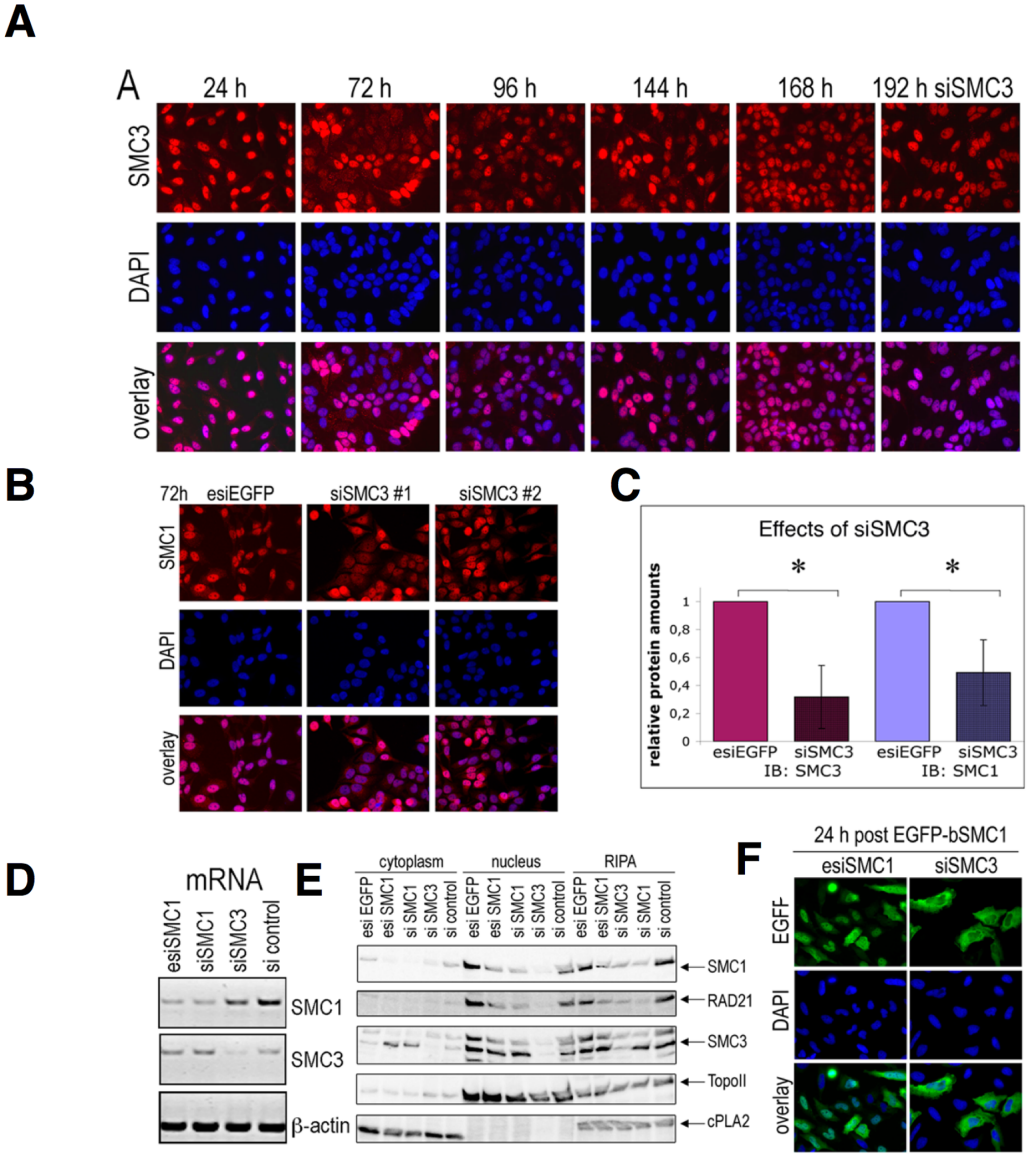
SMC3 knock-down by siRNA reduces the stability of SMC1. (**A**) Time course of reduction and recovery of SMC3 analyzed by IF microscopy with anti-SMC3 (in red) and DAPI (in blue). (**B**) IF analysis using antiSMC1 antibody (in red) and DAPI (in blue). (**C**) Quantification of three independent IB experiments using total cell extracts (RIPA) after siSMC3 treatment. (**D**) Expression of mRNA of Smc1, Smc3 and ß-actin 72 h after esiSMC1, siSMC1, or siSMC3 treatment, compared to control (esiEGFP), was examined by RT PCR and agarose gel electrophoresis. (**E**) Cytoplasmic, nuclear and total cell extracts (RIPA) were prepared 72 h after transfection with esiEGFP, esiSMC1, siSMC1, siSMC3, or non targeting siRNA control. Protein amounts were analyzed by IB using anti-SMC1, anti-RAD21 and anti-SMC3 antibodies. Topo II was used as a nuclear control and cPLA2 as a cytoplasmic loading control. (**F**) 48 h after siSMC3 or esiSMC1 treatment the cells were transfected with EGFP-bSMC1 for further 24 h. The localization of EGFP-bSMC1 was analyzed by IF microscopy using anti-EGFP antibody.

Considering the SMC3 mis-localization reported above for esiSMC1 treated cells, we investigated whether SMC3 knock-down has a similar effect on SMC1. Two different SMC3 siRNAs were tested and behaved similarly. The kinetics of reduction and recovery of SMC3 following siSMC3 #1 was analyzed in detail by IF using anti-SMC3 (Fig. 6A) and IB from total RIPA extracts (Fig. S3A) and are comparable to that of SMC1 knock-down. Similar to the effects of SMC1 knock-down, cell cycle progression was not affected by siSMC3 treatment (Fig. S3A, B). IF analysis using anti-SMC1 antibody revealed loss of SMC1 following siSMC3 treatment (Fig. 6B). Quantification of IB analysis confirmed knock-down of SMC3 by three-fold and the parallel loss of SMC1 to about half (Fig. S3A, Fig. 6C), whereas the mRNA of SMC1 is only mildly affected (Fig. 6D). Further, cytoplasmic, nuclear and total cell extracts obtained 72 h after transfection with esiEGFP, esiSMC1, siSMC1, siSMC3 or an unspecific siRNA control were analyzed by IB using anti-SMC1, anti-RAD21 and anti-SMC3 (Fig. 6E). The results demonstrate specific effects upon SMC3-siRNA treatment on SMC1 and RAD21, which become instable. Thus, in contrast to SMC1, which controls SMC3 localization but not stability, SMC3 is required for SMC1 stability.

Expression of EGFP-bSMC1 for 24 h upon SMC3 knock-down (72 h) did not rescue the loss of endogenous SMC1, and the EGFP-bSMC1 localized in the cytoplasm of siSMC3-treated cells, but in the nucleus and cytoplasm of esiSMC1-treated cells (Fig. 6F). We therefore hypothesize that SMC3 is involved in SMC1 nuclear import.

### Rescue of siSMC3-induced phenotypes by expression of EGFP-msSMC3

**Figure 7:**
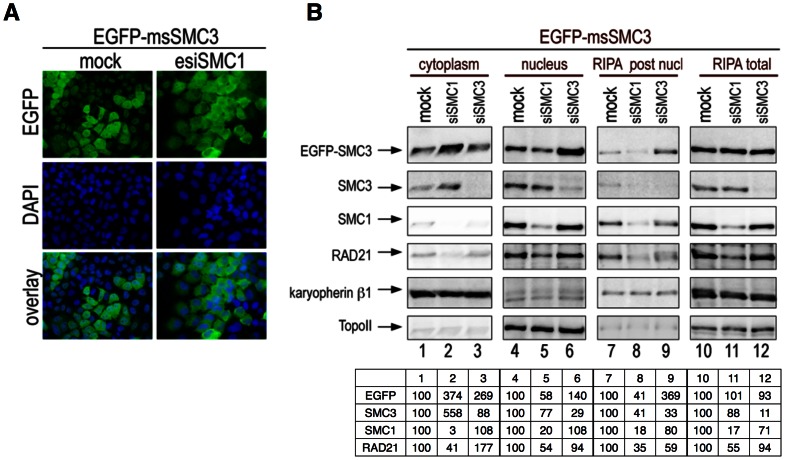
Two-species system: knockdown of human SMC3 by siRNA and rescue by stably expressed EGFP-msSMC3. (**A**) Cells stably transfected with EGFP-msSMC3 were analyzed by IF microscopy. (**B**) Total cell (RIPA), cytoplasmic and nuclear extracts from cells stably expressing EGFP-msSMC3 collected 72 h after treatment with siSMC1 or siSMC3 were analyzed by IB using anti-EGFP, -SMC3 and -SMC1 antibodies. RIPA buffer was used for the final extraction step after the nuclear extraction with 250 mM ammonium sulfate. Topo II and Karyopherin ß1 were used as a loading control. Relative protein levels are shown at the bottom (representative of 3 experiments).

The above data showed that a two-species system functions in complementing effects of the SMC1 knock-down. To rescue the SMC1 instability caused by hSMC3 knock-down, we used a HeLa cell line that stably expresses EGFP-tagged mouse SMC3 (EGFP-msSMC3). Using anti-EGFP in IF (Fig. 7A, mock treated cells) and IB (Fig. 7B, lane 1) we observed an about equal cytoplasmic and nuclear distribution of EGFP-msSMC3. The endogenous SMC3, similar to SMC1, remains normally localized in the nucleus (Fig. 7B, lane 4). Cytoplasmic EGFP-msSMC3 was 4-fold increased following siSMC1 treatment (Fig. 7A and 7B, lane 2), in agreement with the above data from esiSMC1 experiments. Because a large portion of endogenous hSMC3 is also distributed to the cytoplasm upon SMC1 knock-down (Fig. 7B, lane 2; 5-fold increase), SMC1 knock-down similarly affects both, hSMC3 and EGFP-msSMC3. Upon human SMC3 knock-down by siRNA to about 50%, the nuclear EGFP-msSMC3 was app. 1.5-fold increased (Figure 7B, lane 6). Thus, EGFP-msSMC3 is not affected by human siSMC3 and replaces the reduced hSMC3. Moreover, this replacement is functional since it prevented the degradation of endogenous human SMC1 and RAD21 (Figure 7B, lane 6).

Furthermore, RIPA extraction of nuclei after their initial salt extraction revealed that EGFP-msSMC3 did bind with high affinity to chromatin only after siSMC3 treatment (Figure 7B, lane 9), confirming EGFP-msSMC3 functionally replaces hSMC3. Since in the most tightly chromatin-bound fraction, SMC1 and SMC3 exist as heterodimers, and SMC1 is present in the post nuclear RIPA fraction (lane 9), EGFP-msSMC3 became part of that complex.

### Dynamics of Nuclear Cohesin Recovery

**Figure 8:**
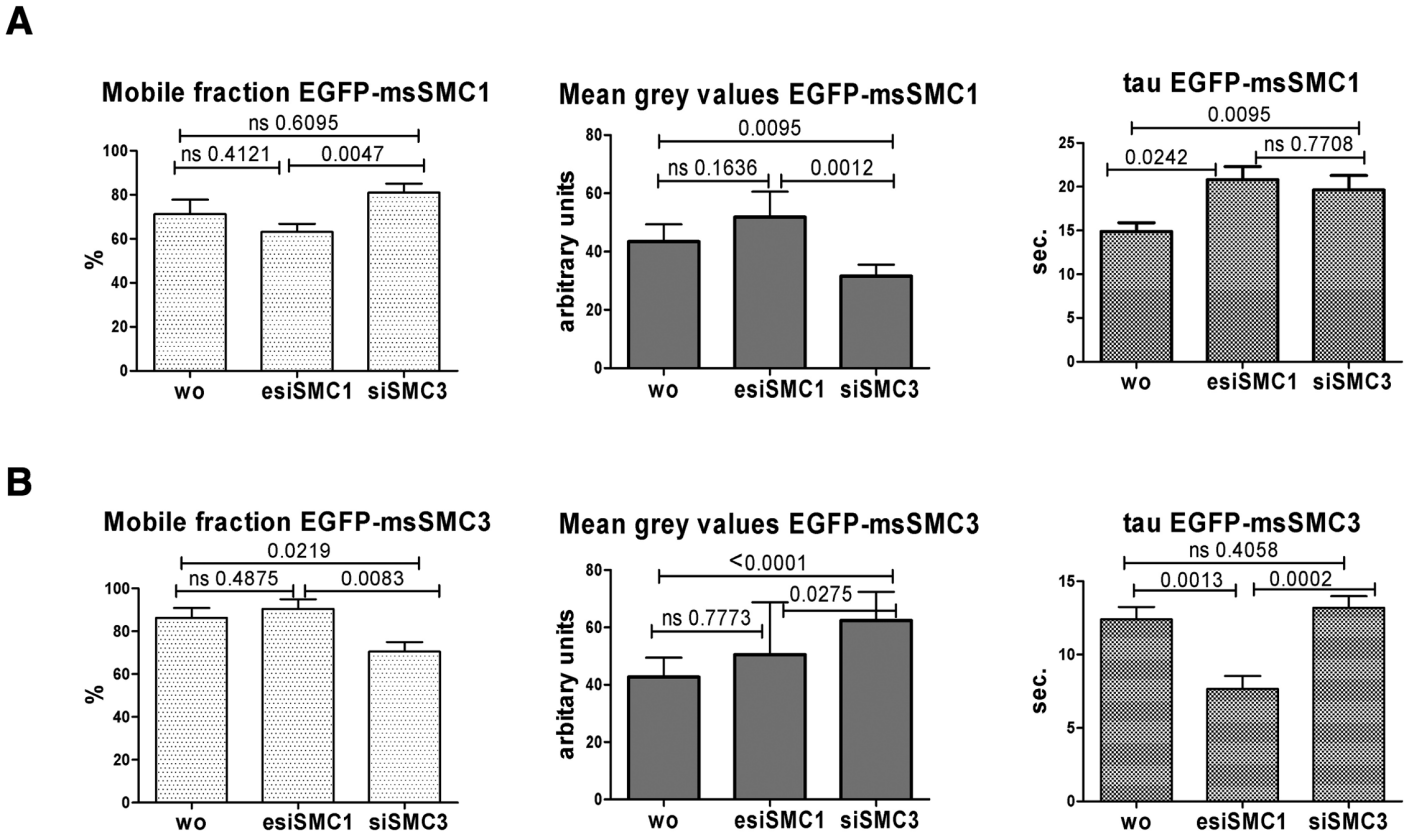
FRAP experiments using EGFP-msSMC1 (A) or EGFP-msSMC3 (B) as bleach substrates. The mobile fraction (recovery), the intensity of the EGFP-tagged protein signal (given as mean grey values), and tau (τ) as a value inversely correlating with mobility are shown; ns  =  non significant; p-values are shown within each graph.

To assess the dynamics of EGFP-msSMC1 in HeLa cells, treated with human esiSMC1, siSMC3, or left untreated, we used FRAP (fluorescence recovery after photobleaching) (Fig. 8A). After a circular region with a diameter of 6 µm of individual nuclei for 1.25 s, the fluorescence recovery was measured for 80 s and reached, in average, levels between 60 and 80% of starting fluorescence. When compared to data from figure 3, this suggests that the mobile fraction corresponds to the pools extracted with 0 mM, 50 mM and partially 250 mM AS, while the immobile Fraction corresponds to some of the 250 mM AS extracts and the RIPA extracts.

The intensity of after recovery of EGFP-msSMC1 was significantly different between cells were treated with either esiSMC1 or siSMC3 in that reduction of endogenous SMC1 allowed more EGFP signal to reappear than reduction of SMC3. Having insufficient amounts of SMC3 available restricts the presence of EGFP-msSMC1, consistent with the instability of SMC1 after SMC3 knockdown. The velocity constant τ as an indicator for the mobility of the EGFP-bSMC1 protein was found increased after esiSMC1 and after siSMC3 treatment, which indicates reduced mobility. In the absence of endogenous SMC1 or SMC3, the transgenic msSMC1 may become more firmly associated with chromatin.

Similarly, the dynamics of EGFP-msSMC3 in HeLa cells was analyzed. Signal recovery after bleaching was 60 to 80% here as well. In contrast to EGFP-msSMC1, the intensity of EGFP-msSMC3 increased most if endogenous SMC3 was decreased, which indicates replacement of the endogenous SMC3 by the msSMC3. The mobile fraction of msSMC3 was lowest in siSMC3 treated cells, suggesting that the msSMC3 became incorporated into the more tightly chromatin-associated cohesion fraction. Without much of an SMC1 partner available in the esiSMC1-treated cells, the amount of EGFP-msSMC3 monomers was increased, resulting in enhanced mobility.

## Discussion

The SMC1/SMC3 heterodimer constitutes the essential backbone of the ring-like cohesin complex. The two SMC proteins are present in an 1∶1 stoichiometry and supposedly do not exist on their own or outside the nucleus in any significant or biologically meaningful manner. It was unclear, however, how the behavior of each of these SMC proteins depends on the presence of the other. To elucidate that dependency we set out to manipulate the balance between these SMC proteins by individually reducing their presence in human cells. Unexpectedly, these studies revealed specific and different effects on each of the SMC proteins.

In particular, we show that (1) under conditions of SMC1 scarcity, SMC3 remained stable and heavily accumulated in the cytoplasm without SMC1 or RAD21 associated; (2) the excess SMC3 that stayed in the SMC1-reduced nucleus is not or only very weakly chromatin-associated; (3) with SMC3 deprived, SMC1 became highly instable; (4) the remaining endogenous SMC1 or SMC3 in the respective knock-down experiments associated with its SMC partner and with RAD21 and constituted the high-affinity chromatin associated fraction; (5) expression of bovine or mouse SMC1 or SMC3 rescued the phenotypes seen upon paucity of endogenous human SMC1 or SMC3; (6) transiently overexpressed SMC1 or stably expressed SMC3 in otherwise untreated cells mostly mis-localized to the cytoplasm, to be rescued only by down-regulation of endogenous SMC1 or SMC3. Decreased mobility of EGFP-tagged SMC3 in case of reduced levels of endogenous SMC3 as seen in FRAP experiments fit to this notion as the EGFP-SMC3 replaces endogenous SMC3 in the tightly chromatin-associated fraction. Similarly, the highly mobile fraction of EGFP-SMC1 decreased when endogenous SMC1 was down-regulated. When SMC3 was reduced, the mobile fraction of EGFP-SMC1 became instable, was degraded due to lack of a partner, and the tightly chromatin-associated fraction increased relatively, explaining the decrease in EGFP-SMC1 mobility upon SMC3 knock-down.

Consistent with these observations, nuclear SMC1 was more stable than mis-localized cytoplasmic SMC1, and excess exogenous SMC3 supports nuclear localization of excess SMC1 under conditions of reduced endogenous SMC3.

In addition, we observed RAD21 degradation whenever there was a shortage of one of the two SMC proteins. Thus, RAD21 is quickly degraded, when it cannot be incorporated into the cohesin complex. At least in SMC1-reduced cells, mRNA levels of Rad21 were increased, possibly in a vain attempt by the cell to compensate for the loss of RAD21 protein. This loss is likely not apoptosis-associated since firstly, the cells did not undergo apoptosis as shown by several assays, and secondly, since the 65/64 kDa C-terminal fragment of RAD21 typically accumulating in apoptotic cells [42], [43] was not observed in immuno blots (data not shown), but would have been detected by the anti-RAD21 antibody, which we used (which recognizes the region between aa 575 to 631).

A further notable result of these studies is that less than 30% of total cohesin is required to maintain proper cell cycle progression and proliferation, and for an unaltered DNA damage response, which includes DNA damage sensitivity, formation of γH2AX and SMC6 foci, and apoptosis. Given the proportion of non-transfected cells in our experiments, we estimate that 10 to 15% of cohesin is sufficient for these important functions of cohesin. This is consistent with previous estimates of a tightly chromosome-associated pool of about one-third of all of cohesin [29]. Whether SMC1 or SMC3 was scarce, the residual SMC1 or SMC3 protein was found largely in the fraction of cohesin that is most tightly associated with chromatin. This is supposedly the essential fraction that provides a minimum of sister chromatid cohesion required for chromosome segregation and cell proliferation. Thus, formation of this pool is predominant at each cell cycle. Obviously, a cell that would not form this pool would die, and thus in cultures suffering from SMC1 or SMC3 deprivation, one selects for cells that maintain this essential cohesin pool. However, since we did not observe increased apoptosis or disturbances in cell division, we assume that the essential cohesin pool is formed first from the available cohesin molecules. One may speculate that a fraction of cohesin may be quite stable, at least sufficiently stable for the few days of treatment and analysis. This would imply that 10 to 15% of cohesin is sufficient to support up to five cell divisions.

The differences in stability of SMC1 and SMC3, if left without a partner, is striking and shall be further analyzed. In preparations of SMC1/SMC3 dimers from calf thymus, we observed frequently a 110–120 kDa polypeptide [44], which was identified as a proteolytic fragment of the app. 160 kDa SMC1, but we never observed an SMC3 degradation product. While highly speculative at this point, one may entertain the idea of a biologically relevant difference in proteolytic sensitivity, which would allow the cell to remove SMC1 under certain, perhaps pathological, conditions.

The high evolutionary conservation of SMC1 and SMC3 within mammals allowed us to establish a two-species system, in which either bovine or mouse SMC1 or SMC3 replaces the respective endogenous human SMC protein in fully functional manner. Also, the EGFP tag placed onto the C-terminus of either bSMC1 or msSMC3 did not visibly interfere with their functions. Rather, the tagged proteins behaved as expected in terms of localization, complex formation and mobility in FRAP assays. Such two-species-systems lend themselves now to structure-function studies of SMC proteins. Under conditions employed throughout these experiments – with knock-down to about 25 or 30% of the individual SMC protein and use of non-synchronized cells – we avoided gross effects on cell cycle progression, cell viability, and DNA repair, which under more forced conditions can be observed in mammalian cells [40], [41], [45], [46]. This allowed us to study the behavior of endogenous or exogenous SMC proteins in vital cells. The knock-down applied here was transient, for after about six days, protein levels were back to starting levels.

Changes in SMC1 or SMC3 protein levels that may occur in human pathologies possibly cause chromosome instability in human colorectal cancers [34] or to chromosomal aberrations [47], [48]. Overexpression of SMC3 in fibroblasts may cause cell transformation [49]. In several pathological human tissues including colon carcinoma and liver metastatic cancer cells high transcript levels of SMC3 were reported [49], [50]. A gene dosage effect of meiosis-specific cohesins REC8 and SMC1ß was recently observed by us in oocytes and spermatocytes [51]. It is too early to hypothesize about physiological functions of individual SMC1 or SMC3 proteins, and the aberrant behavior seen in our experiments would rather argue for pathological roles. For the related SMC protein SMC5, which acts within the SMC5/SMC6 heterodimer in DNA repair, roles in mitotic progression and maintenance of sister chromatid cohesion independent of dimerization with SMC6 were proposed [52]. Mutations in SMC1 or SMC3, which affect the stability of dimerization such as mutations in their hinge domains, could alter the stability and localization of the heterodimer partner. If the dimer dissociates more easily, SMC1 may be degraded and SMC3 localized to the cytoplasm with potential pathological consequences. Similarly, mutations that increase DNA affinity of hinge dimers as described for certain CdLS-type mutations, may reduce dissociation of cohesin from chromatin [26].

The data presented here also call for caution in interpretation of cohesin knock-down experiments. The reduction of either SMC1 or SMC3 triggers very different phenotypes with respect to their cohesin partners. Depending on the particular phenotypes studied, there can be significant consequences of massive cytoplasmic SMC3 accumulation or of SMC1 degradation, for example. Thus, one cannot simply derive firm conclusions on specific biological effects just by depriving one of the cohesin subunits. Ideally, in such experiments SMC1, SMC3 and RAD21 should be individually reduced in parallel and the specific phenotypes compared.

Similarly, overexpression of SMC1 or SMC3 without parallel reduction of endogenous protein causes mis-behavior of the protein. This is in agreement with earlier observations, where over-expressed Smc3p in *S. cerevisiae* or over-expressed SMC1/SMC3 in insect cells were seen in high amounts in cytosolic fractions [10]. In mammalian cells, SMC1 or SMC3 overexpression also causes multipolar spindles [53]. SMC1 and SMC3 both feature bipartite nuclear localization signals [54]. Yet, EGFP-bSMC1 or EGFP-msSMC3 overexpressed in otherwise untreated cells do not properly localize to the nucleus. Only if through knock-down of endogenous SMC1 or SMC3 a more fitting balance between total levels of SMC1 and SMC3 proteins is approached, is nuclear localization achieved. Thus, the accurate balance between SMC1 and SMC3 is essential for their proper localization.

## Supporting Information

Figure S1 **Kinetics of SMC1 knock-down after transient transfection 750 ng/mL esiSMC1 into 1×10^6^ HeLa cells (A).** Total RIPA cell extracts were analyzed by IB using anti-SMC1 and anti-SMC3 antibodies and confirm the specific SMC1 reduction due to esiSMC1, starting at 24 h post transfection. The percentages of SMC1 protein reduction compared to mock treated cells and normalized to unaltered SMC3 protein levels are indicated below. (**B**) Proliferation of cells harvested 72 h after esiSMC1 treatment (as described in A) and analyzed by FACS using CFSE staining. (**C**) The cell cycle status of cells treated as in A) was measured by FACS using propidium iodine. Cells treated with 0.1 µg/mL of mitomycine C (MMC) for 2 h and cultured for additional 24 h served as positive controls for cell cycle arrest. Quantification of the values for G1, S and G2 phases are indicated on the right. (**D**) IF staining of cells either mock-treated or treated for 2 h with 0.1 µg/mL of mitomycine C (MMC) and cultured for either 24 h or 48 h as indicated. Cells were also treated with control esiRNA (esiEGFP) or esiSMC1 RNA, and stained for SMC3, γH2AX and p957-SMC1. (**E**) IF staining for SMC6 of cells treated for 72 h with control esiRNA (esiEGFP) or esiSMC1 and subsequently treated for 2 h with 0.1 µg/mL of mitomycine C (MMC) and cultured for 48 h.
(TIFF)

Figure S2 **Long term cell cycle studies (A) were performed from cells collected 72 to 268 h after esiSMC1 or esiEGFP transfection (as described in S1A).** As positive control, MMC treated cells (1 µg/mL for 2 h and released for 24 h) are included. A second control used untreated cells mixed with MMC treated (mix) cells in a 1∶10 ratio to indicate small changes in cell cycles. The values of G1, GS and G2 phases are summarized in the graph below. (**B**) IB analysis of cells treated with siSMC1 (50 pmol siSMC1/mL and 1×10^6^ cells) for 24 to 120 h compared to esiSMC1 and esiEGFP treated cells (72 h). The membrane was probed with anti-SMC3, re-probed with anti-SMC1, and then re-probed with anti-RAD21 antibodies. The percentages of SMC1 and RAD21, normalized to levels of karyopherin ß1, and compared to esiEGFP treated cells are indicated at the bottom.
(TIFF)

Figure S3 **Kinetics of SMC3 knock-down after siSMC3 treatment, analyzed by IB using anti-SMC3 antibody (A).** The membrane was successively reprobed with anti-SMC1, anti-RAD21 and anti-karyopherin ß1 antibodies. The percentages of protein levels that were normalized to karyopherin ß1 and compared to esiEGFP cells are indicated below. (**B**) A representative cell cycle analysis is shown for cells collected 72 h after esiRNA or siRNA treatment. (**C**) Quantification of the cell cycle status of cells treated with esiRNA or siRNA (three independent experiments).
(TIFF)
