# Supplementary figures and images for: Imbalance of SMC1 and SMC3 Cohesins Causes Specific and Distinct Effects
Source: PLoS One. 2013 Jun 12;8(6):e65149. doi: 10.1371/journal.pone.0065149 (PMC3680458; doi:10.1371/journal.pone.0065149)

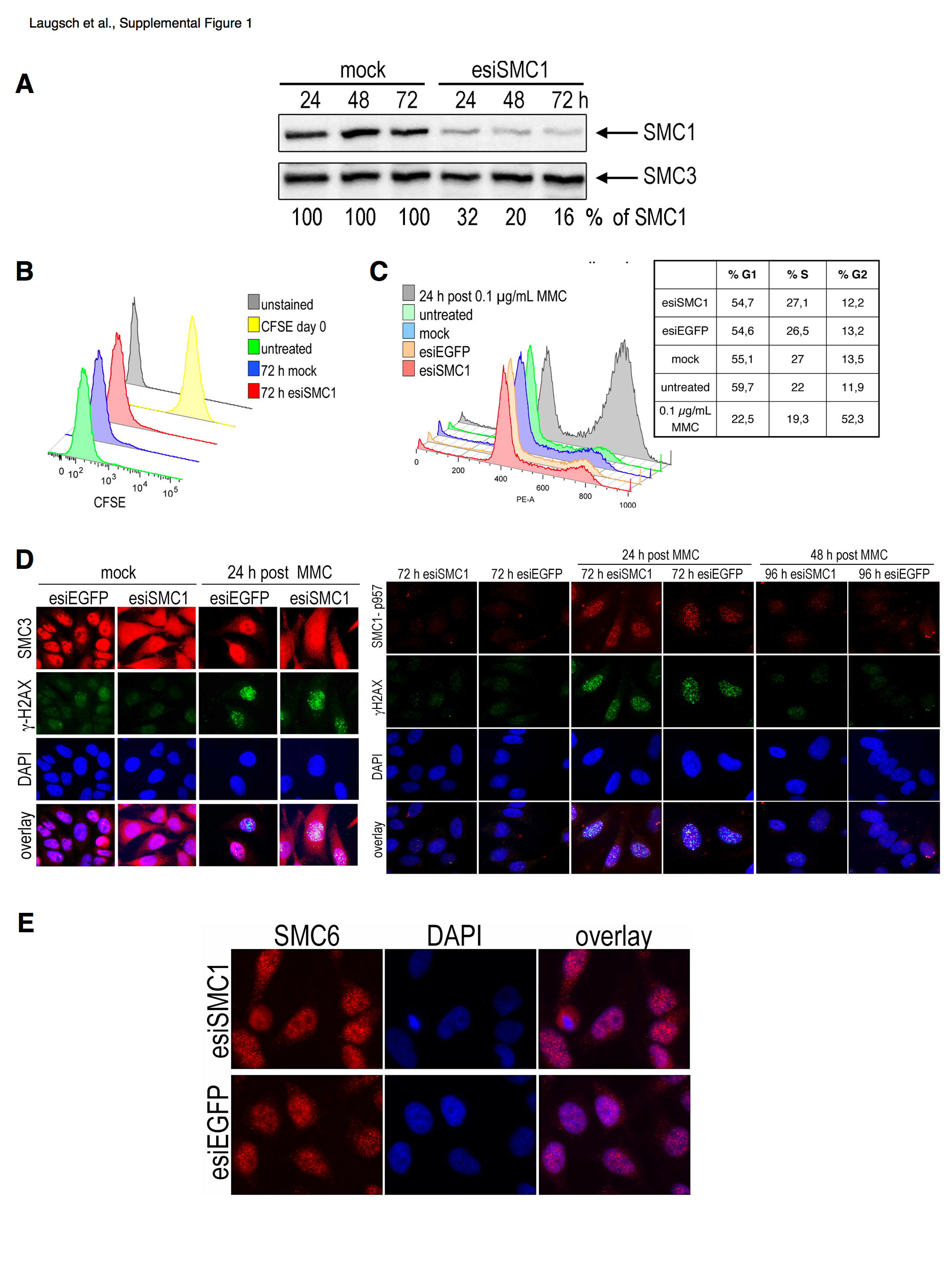

Supplement: Figure S1 — Kinetics of SMC1 knock-down after transient transfection 750 ng/mL esiSMC1 into 1×10^6^ HeLa cells (A). Total RIPA cell extracts were analyzed by IB using anti-SMC1 and anti-SMC3 antibodies and confirm the specific SMC1 reduction due to esiSMC1, starting at 24 h post transfection. The percentages of SMC1 protein reduction compared to mock treated cells and normalized to unaltered SMC3 protein levels are indicated below. (B) Proliferation of cells harvested 72 h after esiSMC1 treatment (as described in A) and analyzed by FACS using CFSE staining. (C) The cell cycle status of cells treated as in A) was measured by FACS using propidium iodine. Cells treated with 0.1 µg/mL of mitomycine C (MMC) for 2 h and cultured for additional 24 h served as positive controls for cell cycle arrest. Quantification of the values for G1, S and G2 phases are indicated on the right. (D) IF staining of cells either mock-treated or treated for 2 h with 0.1 µg/mL of mitomycine C (MMC) and cultured for either 24 h or 48 h as indicated. Cells were also treated with control esiRNA (esiEGFP) or esiSMC1 RNA, and stained for SMC3, γH2AX and p957-SMC1. (E) IF staining for SMC6 of cells treated for 72 h with control esiRNA (esiEGFP) or esiSMC1 and subsequently treated for 2 h with 0.1 µg/mL of mitomycine C (MMC) and cultured for 48 h. (TIFF) [file pone.0065149.s001.tiff]

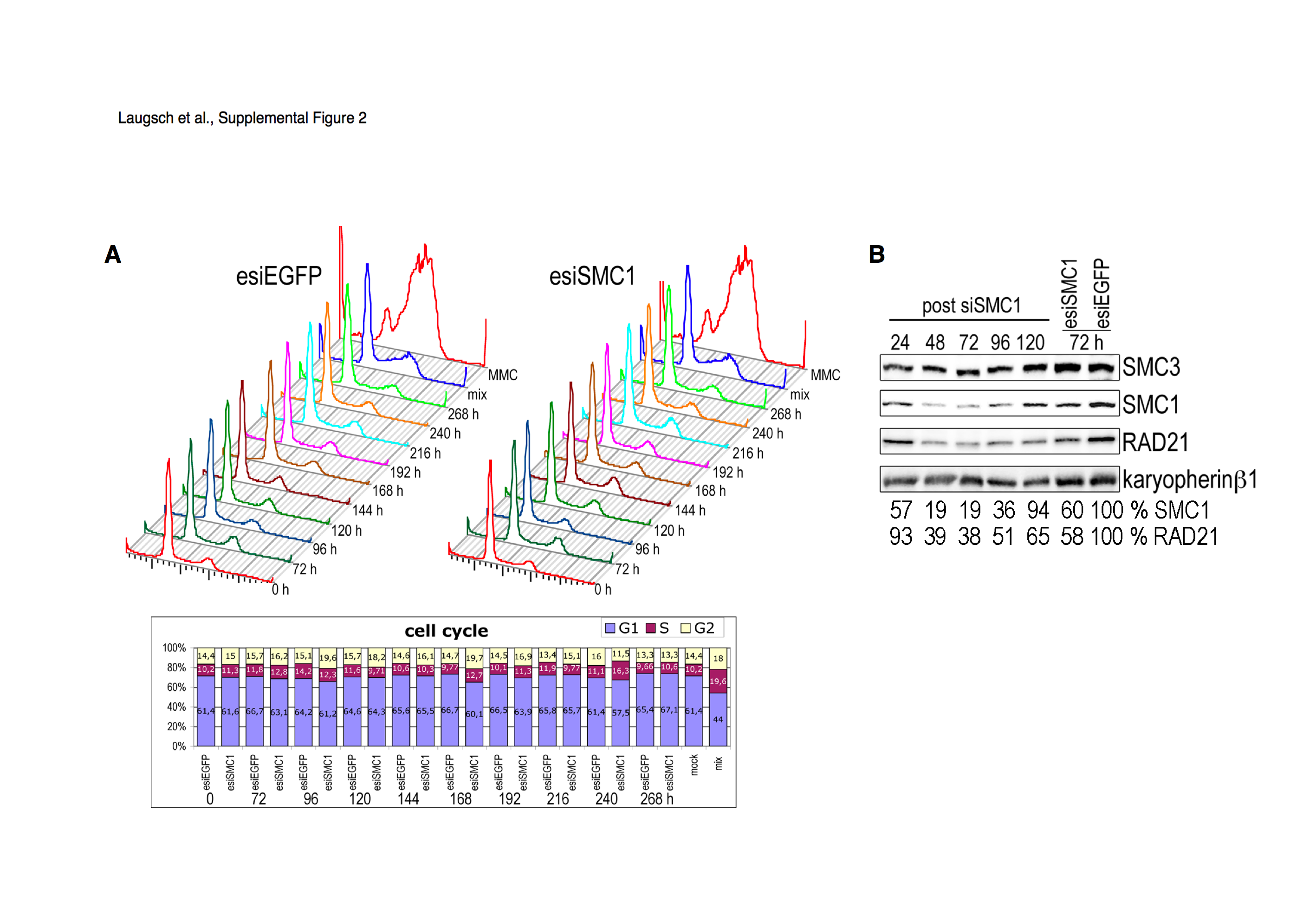

Supplement: Figure S2 — Long term cell cycle studies (A) were performed from cells collected 72 to 268 h after esiSMC1 or esiEGFP transfection (as described in S1A). As positive control, MMC treated cells (1 µg/mL for 2 h and released for 24 h) are included. A second control used untreated cells mixed with MMC treated (mix) cells in a 1∶10 ratio to indicate small changes in cell cycles. The values of G1, GS and G2 phases are summarized in the graph below. (B) IB analysis of cells treated with siSMC1 (50 pmol siSMC1/mL and 1×10^6^ cells) for 24 to 120 h compared to esiSMC1 and esiEGFP treated cells (72 h). The membrane was probed with anti-SMC3, re-probed with anti-SMC1, and then re-probed with anti-RAD21 antibodies. The percentages of SMC1 and RAD21, normalized to levels of karyopherin ß1, and compared to esiEGFP treated cells are indicated at the bottom. (TIFF) [file pone.0065149.s002.tiff]

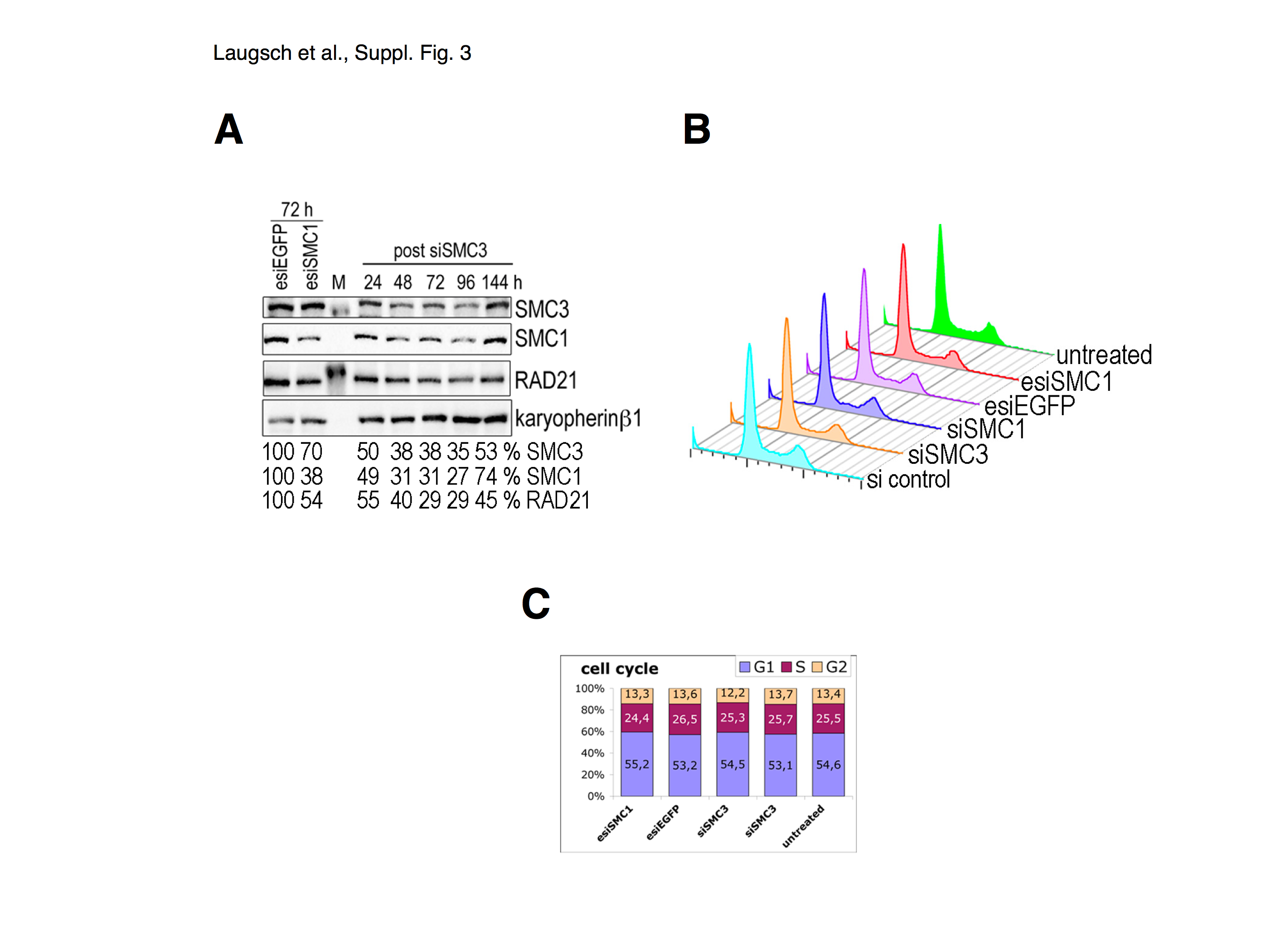

Supplement: Figure S3 — Kinetics of SMC3 knock-down after siSMC3 treatment, analyzed by IB using anti-SMC3 antibody (A). The membrane was successively reprobed with anti-SMC1, anti-RAD21 and anti-karyopherin ß1 antibodies. The percentages of protein levels that were normalized to karyopherin ß1 and compared to esiEGFP cells are indicated below. (B) A representative cell cycle analysis is shown for cells collected 72 h after esiRNA or siRNA treatment. (C) Quantification of the cell cycle status of cells treated with esiRNA or siRNA (three independent experiments). (TIFF) [file pone.0065149.s003.tiff]
